# Association between pilonidal sinus disease and depression: a population-based cohort study

**DOI:** 10.1007/s11136-025-04056-0

**Published:** 2025-09-18

**Authors:** Andreas Krieg, Ernst W. Kolbe, Jan H. Wieltsch, Sabine Leerhoff, Sarah Krieg, Karel Kostev

**Affiliations:** 1 Department of General and Visceral Surgery, Thoracic Surgery and Proctology, Medical Campus OWL, University Hospital Herford, Ruhr University Bochum, Schwarzenmoorstr. 70, 32049 Herford, Germany; 2 Department of Inclusive Medicine, University Hospital Ostwestfalen-Lippe, Bielefeld University, 33617 Bielefeld, Germany; 3 Epidemiology, IQVIA, 60549 Frankfurt, Germany

**Keywords:** Pilonidal sinus disease, PSD, Depression, Quality of life, QoL

## Abstract

**Purpose:**

The aim of this study was to evaluate the association between pilonidal sinus disease (PSD) and the subsequent development of depression, addressing an unmet need to examine the psychosocial impact of PSD in a large, representative outpatient cohort in Germany.

**Methods:**

A retrospective cohort study was conducted using data from the IQVIA Disease Analyzer database, which contains anonymized medical records from 1,293 general practices across Germany. The study included patients aged ≥ 18 years diagnosed with PSD (ICD-10: L05) between 2005 and 2022, excluding those with pre-existing psychiatric conditions. Propensity score matching (5:1) was performed to pair PSD patients with individuals without PSD. Depression diagnoses (ICD-10: F32, F33) within five years after diagnosis were assessed using Kaplan-Meier curves and Cox regression analysis.

**Results:**

A total of 11,245 PSD patients (23% women, mean age 35.3 years) were matched to individuals without PSD. The incidence of depression was higher in PSD patients (11.7%) compared to non-PSD cohort (10.5%) over five years (*p* < 0.001). PSD was associated with an increased risk of depression (HR: 1.16; 95% CI 1.09–1.24), with stronger associations observed in women (HR: 1.25; 95% CI 1.11–1.41) and patients > 45 years of age (HR: 1.44; 95% CI 1.26–1.64).

**Conclusion:**

This study demonstrates a significant association between PSD and depression, highlighting the need for integrated treatment approaches that address both physical and psychological aspects of PSD. Future research should further explore causal mechanisms and develop strategies for prevention and treatment of depression in PSD patients.

**Supplementary Information:**

The online version contains supplementary material available at 10.1007/s11136-025-04056-0.

## Introduction

Pilonidal sinus disease (PSD), is defined as acute or chronic inflammation of the subcutaneous fatty tissue, predominantly located in the area of the sacrococcygeal region. PSD was initially described in 1833 by Herbert Mayo [1], and subsequently coined by Richard Manning Hodges in 1880 [2].

The condition manifests predominantly in adolescents and young adults, with a peak incidence typically occurring between the ages of 14 and 25 [3]. Moreover, the incidence of PSD was reported as 26 per 100,000 population, with a steady increase over the last decade [4, 5]. The underlying reasons for this increase are not yet known, but there is a higher risk of developing PSD in males compared to females, with a ratio of 2.2 to 3.1 [4, 5].

The initial clinical presentation of PSD can vary, and the disease can be divided into two stages: the acute and the chronic [6]. An acute pilonidal abscess usually manifests in a similar manner to a localized, superficial abscess at other sites. Chronic abscess cavities often occur with retained hairs and frequently contain chronic draining sinuses.

Depending on the clinical presentation, the therapeutic approach selected, and the patient’s individual wound healing capacity, the treatment of PSD can be prolonged. In the acute abscess-forming condition, initial incision and drainage typically provide rapid symptom relief [7]. Nevertheless, a secondary, definitive surgical intervention is frequently necessary to prevent recurrence.

In contrast, chronic PSD without abscess formation, on the other hand, may necessitate more extensive surgical excision, often accompanied by prolonged wound healing, particularly in cases managed with open wound techniques [8, 9].

PSD wounds have been demonstrated to exert a substantial impact on psychological well-being, particularly in cases characterized by prolonged healing and physical impairment. As demonstrated in the studies by Stewart et al., wound-related pain, immobility, weight changes, and delayed healing have been identified as factors associated with depression and stress [10, 11]. Consequently, PSD should be regarded as a condition that may entail a substantial treatment burden—both physically and psychologically. Specifically, the chronic nature of symptoms, functional impairment, prolonged wound care, and social limitations have the potential to contribute to the development or exacerbation of depressive symptoms in affected individuals.

Despite the existence of numerous studies and a meta-analysis of randomized trials investigating the extent of depression, anxiety, and QoL depending on different treatment methods [12–14], the informative value of most of these studies is limited due to the small number of cases. In addition, none of these studies have examined the association between PSD and depression in comparison to individuals without PSD, and as a function of age. Therefore, the aim of the present study was to investigate the cumulative incidence of depression in patients with PSD compared to a population without this disorder in a large German cohort.

## Methods

### Database

This retrospective cohort study was based on data from the Disease Analyzer database (IQVIA). This database, which has been used in several previous studies focusing on surgically relevant diseases and depression [15, 16], contains anonymous data on diagnoses, prescriptions, and basic medical and demographic data from the computer systems used in office-based practices [17]. The database covers approximately 3,000 office-based practices in Germany. The sampling method for the Disease Analyzer database uses statistics from the German Medical Association to determine the panel design by specialty group, federal state, community size category and age of the physician. The panel of practices included in the Disease Analyzer database has previously been shown to be representative of general and specialist practices in Germany [17].

## Study population

**Fig. 1:**
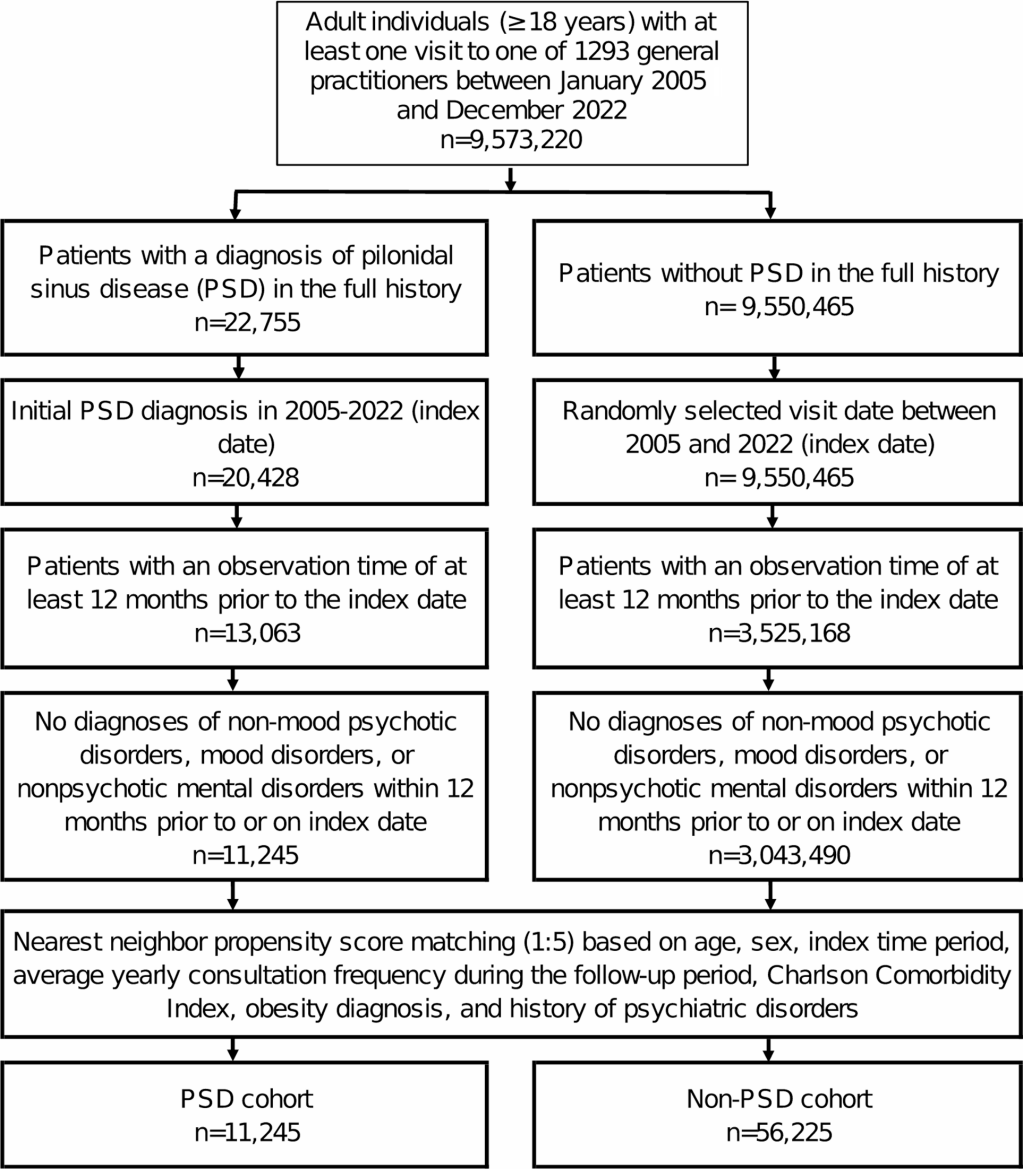
Selection of study patients

The present study comprised patients aged ≥ 18 years who were initially diagnosed with PSD (ICD-10: L05) in 1,293 general practices across Germany between January 2005 and December 2022 (index date; Fig. 1). The index date was defined as the first documented diagnosis of PSD by general practitioners (GPs) between January 2005 and December 2022. Patients with non-mood psychotic disorders (ICD-10: F20-F29), mood disorders (ICD-10: F30-39), or nonpsychotic mental disorders (ICD-10: F40-48) prior to or at the index date were excluded from the study.

Following the application of analogous inclusion criteria, individuals lacking a history of PSD were matched to patients with these specific pathologies using nearest neighbor propensity score matching (5:1) based on age, sex, index time period, average yearly consultation frequency during the follow-up period, and Charlson Comorbidity Index (CCI), obesity diagnosis, as well as a history of psychiatric disorders. For the cohort with non-PSD, the index date was designated as a randomly selected visit between January 2005 and December 2022 (see Fig. 1). A standardized mean difference (SMD) of less than 0.1 was permitted, indicating that adequate covariate balance between the cohorts had been achieved.

## Study outcomes and statistical analyses

The study’s primary objective was to examine the association between depression (ICD-10: F32, F33) and PSD, with a focus on the five-year period following the index date. A comprehensive analysis was conducted using Kaplan-Meier curves to assess the five-year cumulative incidence of depression among individuals with and without PSD. These curves were then compared through the application of the log-rank test. Finally, an univariable Cox regression analysis was conducted to assess the association between PSD and depression. The results of the Cox regression model are displayed as hazard ratios (HRs) and 95% confidence intervals (CIs). Additionally, Cox regression analyses were conducted separately for men and women as well as for four age groups (18–25, 26–35, 36–45, > 45), and separately for PSD with and without abscess (L05.0, L05.9). Due to the multiple comparisons, a *p*-value of less than 0.01 was considered statistically significant. The analyses were carried out using SAS version 9.4 (SAS Institute, Cary, NC, USA).

## Results

### Basic characteristics of the study sample

The present study included 11,245 individuals with PSD (4,030 with abscess and 7,215 without abscess) and matched individuals without these diagnoses (20,150 matched to PSD with abscess and 36,075 matched to PSD without abscess). The basic characteristics of study patients are displayed in Table 1. The mean age was 35.3 years (standard deviation [SD]: 15.7 years), and 23% were women. The patients visited their general practitioners (GPs) an average of 4.9 times per year during the follow-up period. Due to the matched pairs design, no significant differences were observed between the two cohorts in terms of age, sex, visit frequency, CCI, obesity diagnosis, and history of psychiatric disorders (Table 1).

**Table 1:** Baseline characteristics of the study sample (after propensity score matching)

Variable	Proportion among individuals with PSD	Proportion among individuals without PSD	SMD
	*N* = 11,245	*N* = 56,225	
Age (Mean, SD)	35.3 (15.7)	35.3 (15.7)	0.000
Age 18–25	3,834 (34.1)	19,161 (34.0)	
Age 26–35	3,114 (27.7)	15,566 (27.7)	
Age 36–45	1,759 (15.6)	8,805 (15.7)	
Age > 45	2,538 (22.6)	12,693 (22.6)	
Women	2,636 (23.4)	13,214 (23.5)	−0.001
Men	8,609 (76.6)	43,011 (76.5)	
Number of physician visits per year during the follow-up (Mean, SD)	4.9 (4.1)	4.9 (4.1)	−0.004
Charlson Comorbidity Score (Median, IQR)	0 (1)	0 (1)	0.003
CCI 0	5,713 (50.8)	28,626 (50.9)	
CCI 1	3,197 (28.4)	15,976 (28.4)	
CCI 2	1,059 (9.4)	5,270 (9.4)	
CCI ≥ 3	1,276 (11.4)	6,353 (11.3)	
Obesity	282 (2.5)	1,107 (2.0)	−0.005
History of psychiatric disorders	1,080 (9.6)	5,428 (9.7)	−0.001

Proportions of patients in % given, unless otherwise indicated. SD: standard deviation; IQR: interquartile range.

### Association of PSD with subsequent depression diagnosis

**Fig. 2:**
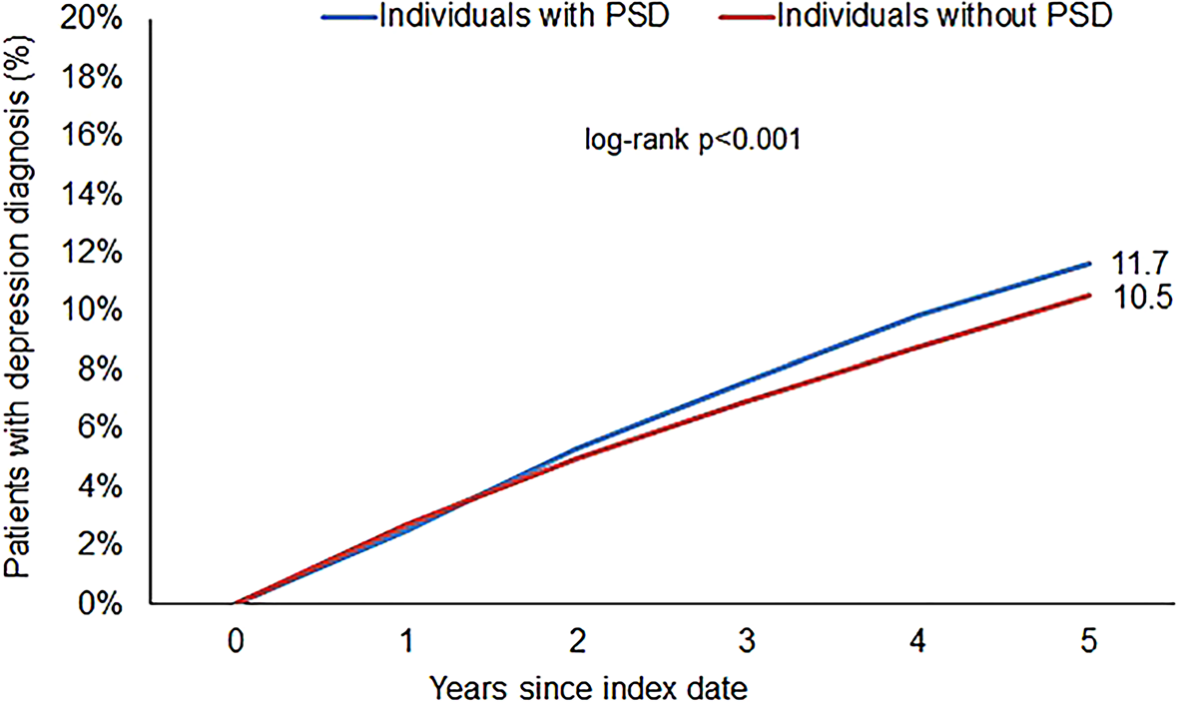
Cumulative incidence of depression in individuals with and without PSD

Following a period of up to five years of observation, 11.7% of patients afflicted with PSD were diagnosed with depression, as compared with 10.5% of the matched cohort devoid of such cysts and sinuses (*p* < 0.001) (Fig. 2). The regression analysis revealed a modest yet statistically significant correlation between PSD and subsequent depression diagnosis (hazard ratio [HR]: 1.16; 95% confidence interval [CI]: 1.09–1.24) (Table 2). Because the initial clinical symptoms of PSD can vary, and the disease has two stages (acute and chronic), we also analyzed these two forms of progression separately. This association remained consistent for both acute PSD with abscess (HR: 1.19; 95% CI 1.07–1.32) and chronic PSD without abscess (HR: 1.15; 95% CI 1.06–1.25). In the age-stratified analysis, the association was stronger in the 36–45-year age group (HR: 1.28; 95% CI 1.01–1.49) and in the > 45-year age group (HR: 1.44; 95% CI 1.26–1.64). Furthermore, the association was more pronounced in women (HR: 1.25; 95% CI 1.11–1.41) compared to men (HR: 1.13; 95% CI 1.04–1.22) (Table 2).

**Table 2:** Association between PSD and subsequent depression in patients followed in general practices in Germany (univariable Cox regression models)

	PSD total		PSD with abscess		PSD without abscess	
Sub-cohorts	HR (95% CI)	*P* value	HR (95% CI)	*P* value	HR (95% CI)	*P* value
Total	1.16 (1.09–1.24)	< 0.001	1.19 (1.07–1.32)	0.002	1.15 (1.06–1.25)	0.001
Age 18–25	0.95 (0.85–1.08)	0.445	1.01 (0.83–1.24)	0.895	0.92 (0.79–1.07)	0.296
Age 26–35	1.15 (1.01–1.30)	0.036	1.23 (1.00-1.51)	0.049	1.10 (0.93–1.29)	0.257
Age 36–45	1.28 (1.01–1.49)	0.002	1.11 (0.85–1.45)	0.434	1.37 (1.14–1.66)	< 0.001
Age > 45	1.44 (1.26–1.64)	< 0.001	1.43 (1.17–1.75)	< 0.001	1.34 (1.22–1.72)	< 0.001
Women	1.25 (1.11–1.41)	< 0.001	1.20 (0.99–1.45)	0.065	1.29 (1.10–1.50)	0.001
Men	1.13 (1.04–1.22)	0.003	1.18 (1.04–1.34)	0.011	1.10 (1.00-1.21)	0.063

## Discussion

In the present study we were able to identify a significant association between PSD and subsequent depression for the first time. The results indicate that patients diagnosed with PSD have an increased incidence of depression by around 16% within a period of five years after diagnosis, compared to a matched cohort without these conditions. PSD can present as an acute abscess or a chronic form without an abscess. An acute abscess typically requires surgical intervention, such as an incision or excision. This is often followed by a phase of secondary wound healing before definitive treatment is performed once the local swelling and inflammatory response have subsided. In contrast, the chronic form offers a range of therapeutic options, including excision with open wound healing, flap procedures, and minimally invasive techniques. Due to these differing clinical courses, we performed a subgroup analysis to investigate whether the association between depression and PSD varies between the form with and without an abscess. However, no significant difference was observed. Furthermore, the risk of developing depression during the observation period was found to be significantly higher in patients over 45 years of age and in women compared to men.

Despite the fact that a considerable number of studies have been conducted on depression, anxiety, and QoL in patients with PSD, these studies have focused exclusively on these psychological disorders and QoL in the context of various treatment modalities in relatively small cohorts [13, 18–22]. It is an irrefutable fact that the optimal treatment for patients afflicted with PSD should facilitate expeditious recovery and guarantee a return to normal daily activities, while concurrently minimizing morbidity. In recent decades, a plethora of surgical procedures for the initial treatment of PSD have been delineated; nevertheless, contemporary practice remains heterogeneous [23]. While certain treatment modalities have yielded superior outcomes for some patients, the complications associated with surgery, particularly those pertaining to wound healing, frequently result in more substantial complications than the underlying disease itself [23]. Various studies on the treatment of PSD have revealed different rates of secondary wound healing after surgery with primary wound closure or flap reconstruction [24–26]. A survey conducted in Germany and ultimately completed by 454 of the 1,191 hospitals contacted revealed that acute PSD was incised in 42% of cases and subsequently treated during the course of a secondary definitive intervention [27]. Chronic PSD was managed in 60% of hospitals by primary excision followed by open wound healing [27]. A flap technique was employed in only 33% of hospitals, while 15% used an off-midline closure procedure [27].

The impact of secondary wound healing is highlighted by research suggesting a significant association between chronic wounds and depression. This is supported by studies demonstrating that patients with chronic wounds are more likely to experience depressive symptoms than healthy controls [28, 29]. The prevalence of moderate to severe depressive symptoms in patients with chronic wounds ranges from 22.1 to 26.6% [29]. Associations between chronic wounds and depression are multifactorial and include factors such as limited mobility, social isolation and chronic pain [30]. In this context, the influence of PSD on the activities of daily living is illustrated by a study that demonstrated that all activities of daily living were negatively impacted by the PSD wound [10].

The association between depression and QoL is well documented [31]. Thus, it has been suggested that this association is bidirectional, i.e. that poor QoL may predispose to the development of depression and vice versa [32]. In fact, people’s QoL improves with the resolution of depressive symptoms, although it does not always reach the level of people without depression [32]. This finding supports the notion that psychosocial stress associated with PSD, such as limited life activity, plays a substantial role in the development of depression. Consequently, the results of our study underscore the necessity to integrate the treatment of depression and the enhancement of QoL into the management of PSD.

As has been demonstrated, pain and depression have been shown to share common pathophysiological mechanisms involving neurotransmitters, neuromodulators and neurohormones [33]. These findings suggest the hypothesis that the association of depression and PSD is probably triggered by the pain associated with this condition. There is a growing body of evidence to suggest that the simultaneous occurrence of chronic pain and depression is associated with interlinked alterations in neuroplasticity [34]. The principal neurobiological mechanisms implicated in both conditions encompass alterations in serotonin levels, pro-inflammatory cytokines, and brain-derived neurotrophic factor [35]. Of particular significance is the bidirectional relationship between pain and depression, which indicates that chronic pain increases the risk of developing depression and vice versa [35].

A comprehensive meta-analysis, which included 65 and 95 articles for the diagnoses of major depression and depressive symptoms, respectively, from 90 different nations, was able to demonstrate that women have a prevalence of depression that is approximately twice as high as that of men over the course of their lives [36]. In line with these observations, the association between PSD and depression was also more pronounced in women than in men in our study.

We also revealed a significantly more pronounced association between PSD and depression in older patients, suggesting a potential link between these conditions. One possible explanation for this phenomenon could be the higher prevalence of chronic comorbidities in older people, as indicated by Makovski et al. in their meta-analysis [37]. These findings suggest that an increase in the number of comorbidities is significantly associated with a reduction in QoL, which in turn has an influence on the development of depression. Furthermore, the often protracted and arduous treatment process for PSD, which involves repeated visits to the doctor, can lead not only to additional physical limitations but also to greater dependence on others due to possible mobility restrictions and an increased sense of loss of meaning. These factors can render older people more vulnerable to depression or exacerbate existing depressive symptoms.

The present study has several notable strengths, including the use of a relatively large and representative cohort and the application of propensity score matching to control for potential confounders. Furthermore, the analysis of data from the Disease Analyzer database makes it possible to study a diverse and heterogeneous patient cohort, thus improving the generalizability of the results. However, it is also necessary to recognize the limitations of the study. Firstly, the study design is retrospective and observational, precluding the establishment of causal relationships. Secondly, diagnoses were based on medical records, which may have sometimes been misclassified or not fully reported. Thirdly, potentially relevant factors—such as the severity of PSD, therapeutic modalities (i.e., open wound healing, flap techniques, minimally invasive techniques), wound healing complications, and socioeconomic status—could not be considered due to data unavailability in the database, which may have influenced the results. Finally, no information was available on the methodology used to diagnose the patient’s depression. This hinders the determination of the diagnostic tool employed, whether it be a questionnaire or another instrument, and the expertise of the treating physician, whether general practitioner or psychiatrist. Moreover, data from hospitals or other specialists for the same patient are not available in the database used, thus preventing the determination of the patient’s medical history or treatment plans in other medical facilities.

In order to establish a causal relationship between PSD and depression, further studies are required that employ high-quality evidence and a prospective, randomized design. Additionally, qualitative studies could assist in gaining a more profound understanding of the psychosocial factors and needs of this patient group. Intervention studies could also contribute to the development of effective strategies for the prevention and treatment of depression in patients with PSD.

## Conclusion

In conclusion, the present study highlights the importance of understanding PSD not only as a physical condition but also as a mental health problem, a crucial aspect that significantly impacts the QoL of those affected. In the context of PSD treatment, healthcare providers should consider the possibility of co-occurring depression and integrate appropriate screening and treatment methods into their practice. Thus, the implementation of a multifaceted care model may have significant potential to reduce the psychosocial burden for those affected. Importantly, further research is warranted to explore the extent to which specific treatment strategies and healing outcomes influence the association between PSD and psychological well-being, particularly depressive symptomatology.

## Supplementary Information

Below is the link to the electronic supplementary material.

Supplementary Material 1

## Data Availability

The data that support the findings of this study are available on request from the corresponding author on reasonable request.

## References

[1] Mayo, H. (1833). Observations on injuries and diseases of the rectum. *Med Chir Rev*, *19*(38), 289–306. PMC5086971 29918031

[2] Hodges, R. (1880). Pilo-nidal sinus. *The Boston Medical and Surgical Journal*, *103*(21), 485–486.

[3] Grabowski, J., Oyetunji, T. A., Goldin, A. B., Baird, R., Gosain, A., Lal, D. R., Kawaguchi, A., Downard, C., Sola, J. E., Arthur, L. G., Shelton, J., Diefenbach, K. A., Kelley-Quon, L. I., Williams, R. F., Ricca, R. L., Dasgupta, R., Peter, S., Sømme, S. D., Guner, S., Y. S., & Jancelewicz, T. (2019). The management of pilonidal disease: A systematic review. *Journal of Pediatric Surgery*, *54*(11), 2210–2221. 30948198 10.1016/j.jpedsurg.2019.02.055

[4] Søndenaa, K., Andersen, E., Nesvik, I., & Søreide, J. A. (1995). Patient characteristics and symptoms in chronic pilonidal sinus disease. *International Journal of Colorectal Disease*, *10*(1), 39–42. 7745322 10.1007/BF00337585

[5] von Oetzmann, C., & Gödeke, J. (2021). Pilonidal sinus disease on the rise: A one-third incidence increase in inpatients in 13 years with substantial regional variation in Germany. *International Journal of Colorectal Disease*, *36*(10), 2135–2145. 33993341 10.1007/s00384-021-03944-4 PMC8426302

[6] Khanna, A., & Rombeau, J. L. (2011). Pilonidal disease. *Clinics in Colon and Rectal Surgery*, *24*(1), 46–53. 22379405 10.1055/s-0031-1272823 PMC3140333

[7] Iesalnieks, I., & Ommer, A. (2019). The management of pilonidal sinus. *Dtsch Arztebl Int*, *116*(1–2), 12–21. 30782310 10.3238/arztebl.2019.0012 PMC6384517

[8] Prassas, D., Rolfs, T. M., Schumacher, F. J., & Krieg, A. (2018). Karydakis flap reconstruction versus limberg flap transposition for pilonidal sinus disease: A meta-analysis of randomized controlled trials. *Langenbecks Arch Surg*, *403*(5), 547–554. 30066108 10.1007/s00423-018-1697-7

[9] Gil, L. A., Deans, K. J., & Minneci, P. C. (2023). Management of pilonidal disease: A review. *JAMA Surg*, *158*(8), 875–883. 37256592 10.1001/jamasurg.2023.0373

[10] Stewart, A. M., Baker, J. D., & Elliott, D. (2011). The effects of a sacrococcygeal pilonidal sinus wound on activities of living: Thematic analysis of participant interviews. *Journal of Clinical Nursing*, *20*(21–22), 3174–3182. 21831106 10.1111/j.1365-2702.2011.03806.x

[11] Stewart, A. M., Baker, J. D., & Elliott, D. (2012). The psychological wellbeing of patients following excision of a pilonidal sinus. *Journal of Wound Care*, *21*(12), 595–596. 23299269 10.12968/jowc.2012.21.12.595

[12] Berthier, C., Bérard, E., Meresse, T., Grolleau, J. L., Herlin, C., & Chaput, B. (2019). A comparison of flap reconstruction vs the laying open technique or excision and direct suture for pilonidal sinus disease: A meta-analysis of randomised studies. *International Wound Journal*, *16*(5), 1119–1135. 31230414 10.1111/iwj.13163 PMC7948539

[13] Salimi-Jazi, F., Abrajano, C., Garza, D., Rafeeqi, T., Yousefi, R., Hartman, E., Hah, K., Wilcox, M., Diyaolu, M., Chao, S., Su, W., Hui, T., Mueller, C., Fuchs, J., & Chiu, B. (2022). Burden of pilonidal disease and improvement in quality of life after treatment in adolescents. *Pediatric Surgery International*, *38*(10), 1453–1459. 35842877 10.1007/s00383-022-05175-2

[14] Duman, K., Ozdemir, Y., Yucel, E., & Akin, M. L. (2014). Comparison of depression, anxiety and long-term quality of health in patients with a history of either primary closure or limberg flap reconstruction for pilonidal sinus. *Clinics*, *69*(6), 384–387. 24964301 10.6061/clinics/2014(06)03 PMC4050323

[15] Kostev, K., Konrad, M., Smith, L., & Krieg, S. (2024). Hemorrhoids are associated with an increased risk of depression in germany: A retrospective cohort study in primary care outpatients. *Journal of Psychiatric Research*, *175*, 381–385. 38772129 10.1016/j.jpsychires.2024.05.028

[16] Krieg, A., Kolbe, E. W., Kaspari, M., Krieg, S., Loosen, S. H., Roderburg, C., & Kostev, K. (2024). *Depression in patients with anorectal fistulas and anal fissures: A propensity score-matched cohort study*. Qual Life Res. 10.1007/s11136-024-03863-1 PMC11919947 39674845

[17] Rathmann, W., Bongaerts, B., Carius, H. J., Kruppert, S., & Kostev, K. (2018). Basic characteristics and representativeness of the German disease analyzer database. *International Journal of Clinical Pharmacology and Therapeutics*, *56*(10), 459–466. 30168417

[18] Esposito, C., Lepore, B., Cerulo, M., Borgogni, R., Del Conte, F., Coppola, V., Di Mento, C., Carulli, R., Cardone, R., Cortese, G., Esposito, G., & Escolino, M. (2023). Quality of life of pediatric patients operated for pilonidal sinus disease. *Eur J Pediatr*, *182*(1), 25–30. 36348071 10.1007/s00431-022-04678-3 PMC9829630

[19] Abdelnaby, M., Fathy, M., Emile, S. H., Arnous, M., Balata, M., Abdelmawla, A., & Abdallah, E. (2021). Sinus laser therapy versus sinus Lay open in the management of sacrococcygeal pilonidal disease. *Colorectal Disease*, *23*(9), 2456–2465. 34042233 10.1111/codi.15755

[20] Meinero, P., La Torre, M., Lisi, G., Stazi, A., Carbone, A., Regusci, L., & Fasolini, F. (2019). Endoscopic pilonidal sinus treatment (EPSiT) in recurrent pilonidal disease: A prospective international multicenter study. *International Journal of Colorectal Disease*, *34*(4), 741–746. 30719564 10.1007/s00384-019-03256-8

[21] Mamaloudis, I., Perivoliotis, K., Zlatanos, C., Baloyiannis, I., Spyridakis, M., Kouvata, E., Samara, A. A., Christodoulidis, G., & Tepetes, K. (2022). The role of alginate dressings in wound healing and quality of life after pilonidal sinus resection: A randomised controlled trial. *International Wound Journal*, *19*(6), 1528–1538. 35043571 10.1111/iwj.13752 PMC9493221

[22] Henry, O. S., Farr, B. J., Check, N. M., & Mooney, D. P. (2021). A minimally invasive pilonidal protocol improves quality of life in adolescents. *Journal of Pediatric Surgery*, *56*(10), 1861–1864. 33279217 10.1016/j.jpedsurg.2020.11.012

[23] Harries, R. L., Alqallaf, A., Torkington, J., & Harding, K. G. (2019). Management of sacrococcygeal pilonidal sinus disease. *International Wound Journal*, *16*(2), 370–378. 30440104 10.1111/iwj.13042 PMC7949345

[24] Baier, P. K., Baumgartner, U., Furtwängler, A., Holzinger, F., & Schöffel, U. (2002). [Therapy of the pilonidal sinus–Primary wound closure or open wound after excision]. *Zentralblatt Fur Chirurgie*, *127*(4), 310–314. 12085282 10.1055/s-2002-31557

[25] Käser, S. A., Zengaffinen, R., Uhlmann, M., Glaser, C., & Maurer, C. A. (2015). Primary wound closure with A limberg flap vs. secondary wound healing after excision of A pilonidal sinus: A multicentre randomised controlled study. *International Journal of Colorectal Disease*, *30*(1), 97–103. 25367184 10.1007/s00384-014-2057-x

[26] Dahmann, S., Lebo, P. B., & Meyer-Marcotty, M. V. (2016). [Comparison of treatments for an infected pilonidal sinus: Differences in Scar quality and outcome between secondary wound healing and limberg flap in a prospective study]. *Handchirurgie, Mikrochirurgie, Plastische Chirurgie*, *48*(2), 111–119. 27096210 10.1055/s-0041-111322

[27] Schneider, R., Dettmer, M., Peters, N., Lamdark, T., Luedi, M., Adamina, M., & Doll, D. (2021). The current status of surgical pilonidal sinus disease therapy in Germany. *European Surgery*, 54.

[28] Fino, P., Di Taranto, G., Pierro, A., Kacjulite, J., Codolini, L., Onesti, M. G., Toscani, M., & Tarallo, M. (2019). Depression risk among patients with chronic wounds. *European Review for Medical and Pharmacological Sciences*, *23*(10), 4310–4312. 31173303 10.26355/eurrev_201905_17936

[29] Zhou, K., & Jia, P. (2016). Depressive symptoms in patients with wounds: A cross-sectional study. *Wound Repair and Regeneration : official Publication of the Wound Healing Society [And] the European Tissue Repair Society*, *24*(6), 1059–1065. 10.1111/wrr.12484 27717087

[30] Kodange, C. (2021). Screening for depression in patients with chronic wounds. *Advances in Skin & Wound Care*, *34*(9), 502–503. 34415256 10.1097/01.ASW.0000767380.11275.88

[31] Sivertsen, H., Bjørkløf, G. H., Engedal, K., Selbæk, G., & Helvik, A. S. (2015). Depression and quality of life in older persons: A review. *Dementia and Geriatric Cognitive Disorders*, *40*(5–6), 311–339. 26360014 10.1159/000437299

[32] Hohls, J. K., König, H. H., Quirke, E., & Hajek, A. (2021). Anxiety, depression and quality of Life-A systematic review of evidence from longitudinal observational studies. *International Journal of Environmental Research and Public Health*, 18(22). 10.3390/ijerph182212022 PMC8621394 34831779

[33] Han, C., & Pae, C. U. (2015). Pain and depression: A Neurobiological perspective of their relationship. *Psychiatry Investig*, *12*(1), 1–8. 25670939 10.4306/pi.2015.12.1.1 PMC4310906

[34] Sheng, J., Liu, S., Wang, Y., Cui, R., & Zhang, X. (2017). The link between depression and chronic pain: Neural mechanisms in the brain. *Neural Plasticity*, *2017*, 9724371. 28706741 10.1155/2017/9724371 PMC5494581

[35] Boakye, P. A., Olechowski, C., Rashiq, S., Verrier, M. J., Kerr, B., Witmans, M., Baker, G., Joyce, A., & Dick, B. D. (2016). A critical review of Neurobiological factors involved in the interactions between chronic pain, depression, and sleep disruption. *Clinical Journal of Pain*, *32*(4), 327–336. 26035521 10.1097/AJP.0000000000000260

[36] Salk, R. H., Hyde, J. S., & Abramson, L. Y. (2017). Gender differences in depression in representative National samples: Meta-analyses of diagnoses and symptoms. *Psychological Bulletin*, *143*(8), 783–822. 28447828 10.1037/bul0000102 PMC5532074

[37] Makovski, T. T., Schmitz, S., Zeegers, M. P., Stranges, S., & van den Akker, M. (2019). Multimorbidity and quality of life: Systematic literature review and meta-analysis. *Ageing Research Reviews*, *53*, 100903. 31048032 10.1016/j.arr.2019.04.005

